# A Triggering Event of Central Retinal Artery Occlusion With Concurrent Ischemic Stroke

**DOI:** 10.7759/cureus.53577

**Published:** 2024-02-04

**Authors:** Lai Zhong Yang, Qi Zhe Ngoo, Vithiaa Nilamani, Rafidah Sudarno

**Affiliations:** 1 Department of Ophthalmology and Visual Sciences, School of Medical Sciences, Universiti Sains Malaysia, Kubang Kerian, MYS; 2 Department of Ophthalmology, Hospital Tengku Ampuan Rahimah, Klang, MYS

**Keywords:** ophthalmology, neurology, ocular emergency, statin, aspirin, smoker, dyslipidemia, stroke, central retinal artery occlusion (crao)

## Abstract

We report a case of central retinal artery occlusion with concurrent ischemic stroke in a young patient. A 34-year-old Malay gentleman, an ex-smoker with underlying dyslipidemia, however, not on medication or follow-up, presented with acute, generalized, and painless right eye blurring of vision for one day. He also complained of on-and-off headaches for the past three months prior to the presentation. Visual acuity assessment demonstrated hand movement in the right eye, whereas in the left eye, it was 6/6, along with a right eye relative afferent pupillary defect. His right eye showed reduced optic nerve function and unremarkable anterior segment, with fundus examination revealing the presence of a cherry red spot, pale macula, boxcarring pattern over superior arcuate, and vascularized retina over inferior optic disc with blurred optic disc margin. The left eye examination was unremarkable. All cranial nerves were intact, except for the optic nerve. He was admitted to the ward. While in the ward, he developed a sudden onset of left-sided upper and lower limb weakness and numbness and was diagnosed with acute ischemic stroke. Blood investigations showed raised low-density lipoprotein cholesterol of 3.51 mmol/L, anti-nuclear antibody (ANA) positive, with electrocardiogram (ECG) sinus rhythm, and no atrial fibrillation. The echocardiogram was normal, and computed tomography angiography of the brain showed non-opacification at the origin and proximal part of the right ophthalmic artery, suspicious of thrombosis with distal reconstitution, with no evidence of thrombosis in the rest of neck and intracranial arteries. The patient was started on aspirin 150 mg once a day and atorvastatin 20 mg at night; subsequently, his vision improved slightly.

## Introduction

Central retinal artery occlusion (CRAO) is classified as an ophthalmic emergency, caused by the acute occlusion of the retinal artery, often resulting in visual impairment [1] due to insufficient oxygen delivery to the retina. The occlusion can result from an embolus or thrombus, traumatic vessel wall damage, spasm, or vasculitis. The first division of the ophthalmic artery gives rise to the central retinal artery, which provides blood supply to the optic nerve fibers and the inner third of the retina. Once it penetrates the eye, the central retinal artery further separates into superior and inferior branches. Originating as a distinct branch from the ophthalmic artery, the short posterior ciliary artery generates a subsidiary branch known as the cilioretinal artery.

Various etiologies have been documented as contributors to retinal artery occlusion, with a predominant emphasis on cardiac origins or emboli arising from the carotid artery. The prevalence of concomitant ipsilateral carotid artery pathologies spans from 3% to 96%, and emboli originating from cardiac sources have been identified in 24-72% of cases [2-5]. The guidelines endorsed by the American Heart Association/American Stroke Association (AHA/ASA) advocate for immediate cerebral imaging in all cases of suspected retinal ischemia [6]. Consequently, interdisciplinary collaboration is imperative to anticipate potential cerebral ischemic events in patients afflicted with CRAO.

The rationale for this case report on a young patient with CRAO concurrent with an ischemic stroke serves as a triggering event that emphasizes the importance of a high level of stroke vigilance in the management of patients with CRAO. Informed consent was obtained from the patient for this case report publication.

## Case presentation

**Figure 1:**
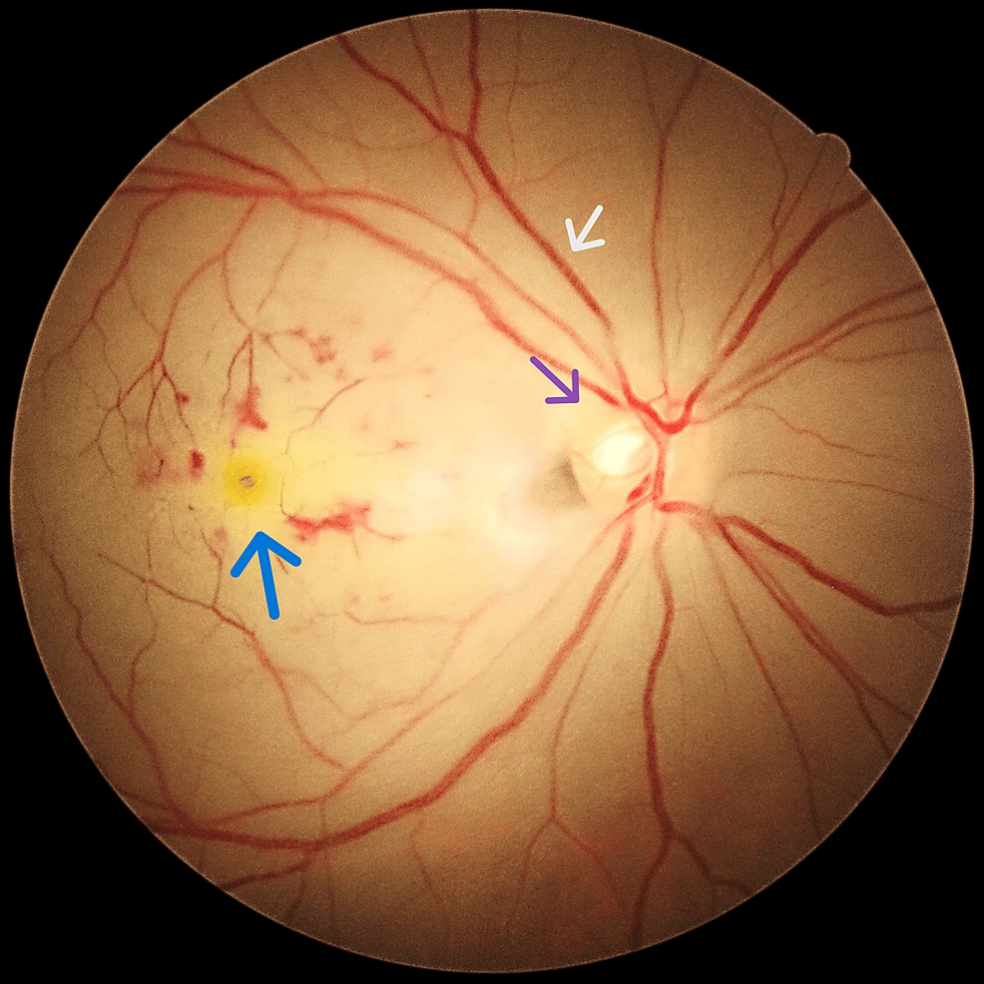
Right eye fundus photo with a cherry red spot (blue arrow), boxcarring (grey arrow), blurred disc margin (purple arrow), and pale macula.

**Figure 2:**
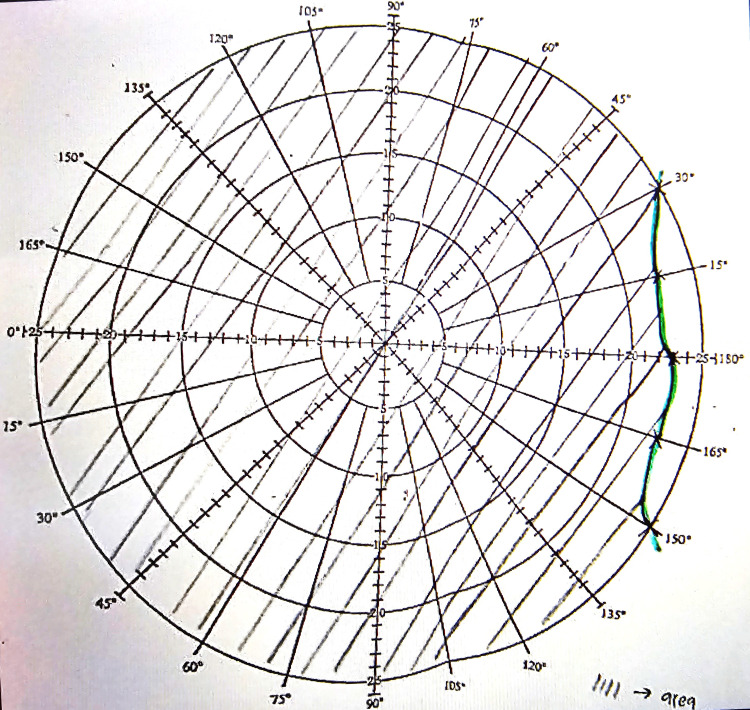
Right eye Bjerrum with visual field loss.

A 34-year-old Malay gentleman, a former smoker with underlying dyslipidemia but currently not on medication, presented with the sudden onset of painless vision blurring in the right eye over a duration of one day. He reported experiencing intermittent headaches for the past three months. Visual acuity assessment demonstrated hand movement in the right eye, whereas in the left eye, it was 6/6, along with a right eye relative afferent pupillary defect. Assessment of the right eye revealed diminished optic nerve function, evidenced by a pale macula with a cherry-red spot, boxcarring over the superior arcuate, vascularized retina over the inferior optic disc, and a blurred optic disc margin (Figure 1). Right eye Bjerrum showed visual field loss (Figure 2).

The left eye examination was unremarkable. Ocular massage was initiated immediately. All cranial nerves were intact except for the optic nerve. He was admitted to the ward for further assessment and optimization due to the young onset of the disease.

While he was in the ward, after three days of admission, he developed a sudden onset of left-sided upper and lower limb weakness and numbness and was diagnosed with an acute stroke. Blood investigations are shown in Table 1.

**Table 1:** Blood parameters of the patient.

Blood parameters	Result	Reference range
Low-density lipoprotein (LDL)	3.51 mmol/l	<2.6 mmol/l
Anti-nuclear antibody (ANA)	>1:80	<1:80
C-reactive protein (CRP)	<0.9 mg/dl	<0.9 mg/dL
Rheumatoid factor	<20 IU/ml	<20 IU/ml
Syphilis rapid plasma reagin	1:8	Non-reactive
Human immunodeficiency virus (HIV) antigen/antibody	Non-reactive	Non-reactive
Hepatitis C antibody	Non-reactive	Non-reactive
Hepatitis B surface antigen	Non-reactive	Non-reactive

The results showed a raised low-density lipoprotein cholesterol of 3.51 mmol/L, anti-nuclear antibody (ANA) positive, C-reactive protein < 0.50 mg/L, negative rheumatoid factor, non-reactive infective screening, including syphilis rapid plasma reagin, HIV antigen/antibody, hepatitis C antibody, and hepatitis B surface antigen, with ECG showing sinus rhythm and no atrial fibrillation. Echocardiogram was normal, and CT angiography of the brain showed non-opacification at the origin and proximal part of the right ophthalmic artery, suspicious of thrombosis with distal reconstitution, with no evidence of thrombosis in the rest of the neck and intracranial arteries. The patient was started on aspirin 150 mg once a day and atorvastatin 20 mg at night; subsequently, his vision improved slightly.

**Figure 3:**
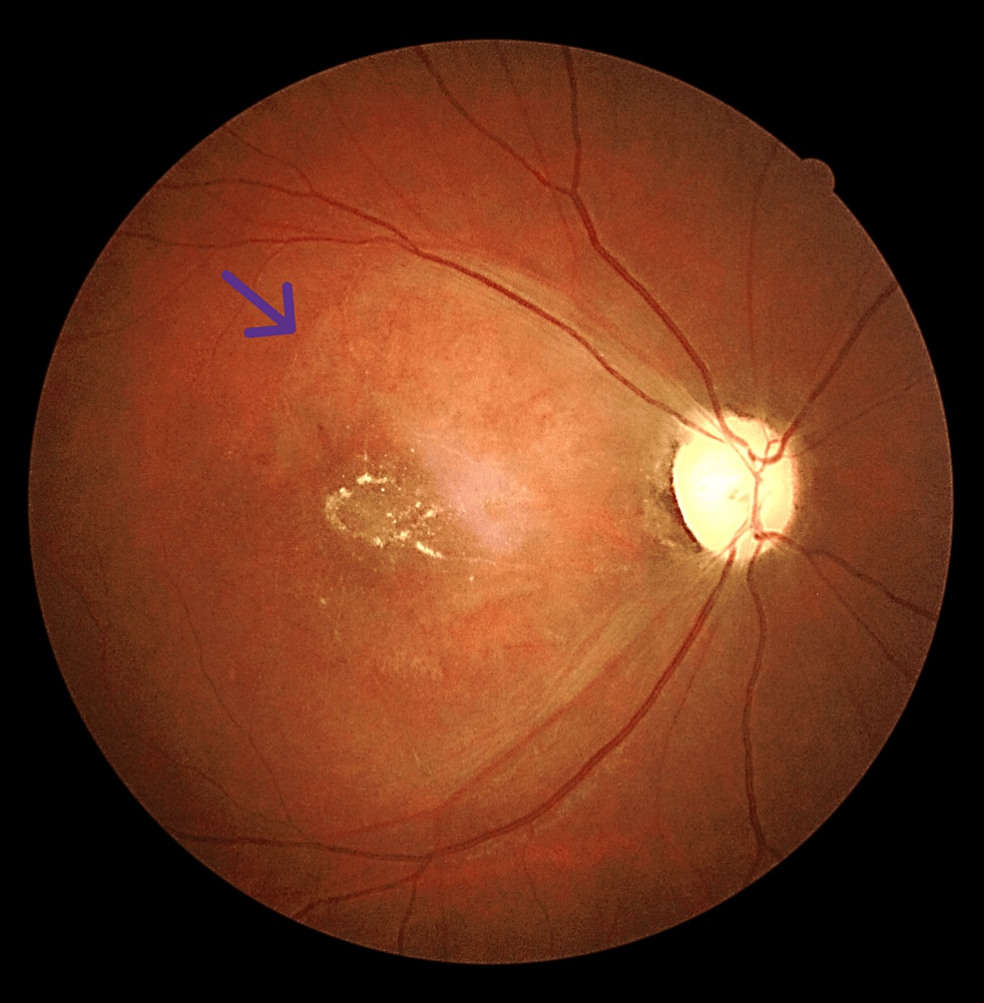
Fundus photo two months after symptom onset showing pale posterior pole (purple arrow).

He came back for a review after two months, with the right eye fundus noted as pale in the posterior pole and optic disc (Figure 3).

## Discussion

CRAO constitutes a critical ophthalmic emergency, necessitating prompt assessment and transfer to a specialized stroke center. This serious condition can be caused by an embolus, thrombus, vasculitis, trauma, or spasm and often presents with concurrent ischemic stroke [7]. This patient experienced an acute cerebral stroke while in the ward, just a few days from the onset of CRAO.

A study demonstrated that there is an increased risk of in-patient stroke (12.9%), acute myocardial infarction (3.7%), and death in CRAO patients, with a combined risk of up to 19% [8]. Moreover, positive predictors of in-patient stroke included female sex, smoking, hypertension, carotid artery stenosis, and alcohol dependence or abuse [8].

In a study, findings suggested that the risk of getting a symptomatic ischemic stroke is 2.2% during the 15-day period preceding and following a CRAO [9]. Additional research, encompassing a systematic review, revealed that 30% of individuals with acute CRAO and 25% of those with acute branch retinal artery occlusion (BRAO) manifested acute cerebral ischemia within a seven-day timeframe [10]. This underscores the importance of promptly referring CRAO patients for neurological evaluation and brain imaging to minimize potential morbidity and mortality.

## Conclusions

In conclusion, comorbidities such as hypertension, hyperlipidemia, and diabetes mellitus are major risk factors for CRAO. The timely detection and control of hypertension and dyslipidemia play pivotal roles in mitigating the risk of CRAO. Hence, early identification and efficient management of these medical conditions are imperative. Additionally, individuals diagnosed with CRAO should receive prompt attention, including immediate management and referral for neurological evaluation and brain imaging. This comprehensive approach ensures optimized care and enhances outcomes for affected patients.

## References

[1] Yuzurihara D, Iijima H (2004). Visual outcome in central retinal and branch retinal artery occlusion. Jpn J Ophthalmol.

[2] Kramer M, Goldenberg-Cohen N, Shapira Y (2001). Role of transesophageal echocardiography in the evaluation of patients with retinal artery occlusion. Ophthalmology.

[3] Inatomi Y, Hino H, Hashimoto Y, Furuyoshi N, Misumi I, Uchino M (2001). Transesophageal echocardiography for detection of cardiac diseases in patients with retinal artery occlusion. Intern Med.

[4] Douglas DJ, Schuler JJ, Buchbinder D, Dillon BC, Flanigan DP (1988). The association of central retinal artery occlusion and extracranial carotid artery disease. Ann Surg.

[5] Arnold M, Koerner U, Remonda L (2005). Comparison of intra-arterial thrombolysis with conventional treatment in patients with acute central retinal artery occlusion. J Neurol Neurosurg Psychiatry.

[6] Furie KL, Kasner SE, Adams RJ (2011). Guidelines for the prevention of stroke in patients with stroke or transient ischemic attack: a guideline for healthcare professionals from the American Heart Association/American Stroke Association. Stroke.

[7] Lee J, Kim SW, Lee SC, Kwon OW, Kim YD, Byeon SH (2014). Co-occurrence of acute retinal artery occlusion and acute ischemic stroke: diffusion-weighted magnetic resonance imaging study. Am J Ophthalmol.

[8] Mir TA, Arham AZ, Fang W, Alqahtani F, Alkhouli M, Gallo J, Hinkle DM (2019). Acute vascular ischemic events in patients with central retinal artery occlusion in the United States: a nationwide study 2003-2014. Am J Ophthalmol.

[9] Chodnicki KD, Tanke LB, Pulido JS (2022). Stroke risk before and after central retinal artery occlusion: a population-based analysis. Ophthalmology.

[10] Fallico M, Lotery AJ, Longo A (2020). Risk of acute stroke in patients with retinal artery occlusion: a systematic review and meta-analysis. Eye (Lond).

